# Methylammonium‐Free Ink for Blade‐Coating of Pure‐Phase α‐FAPbI_3_ Perovskite Films in Air

**DOI:** 10.1002/advs.202410266

**Published:** 2024-10-22

**Authors:** Jianbo Liu, Jingwen Cao, Meng Zhang, Xiaoran Sun, Tian Hou, Xiangyu Yang, Linhu Xiang, Xin Liu, Zhipeng Fu, Yuelong Huang, Feng Wang, Wenhua Zhang, Xiaojing Hao

**Affiliations:** ^1^ School of New Energy and Materials Southwest Petroleum University Chengdu 610500 China; ^2^ The Australian Centre for Advanced Photovoltaics School of Photovoltaic and Renewable Energy Engineering University of New South Wales Sydney New South Wales 2052 Australia; ^3^ Phoenixolar Optoelectronics Co. Ltd Huzhou Zhejiang 313200 China; ^4^ Center for Combustion Energy Department of Energy and Power Engineering Tsinghua University Beijing 100084 China; ^5^ Yunnan Key Laboratory of Carbon Neutrality and Green Low‐carbon Technologies Yunnan Key Laboratory for Micro/Nano Materials & Technology Southwest United Graduate School School of Materials and Energy Yunnan University Kunming 650504 China

**Keywords:** 2‐imidazolidinone, blade‐coating, perovskite ink, perovskite modules, phase‐transition

## Abstract

The α‐c (α‐FAPbI_3_) has been extensively employed in the fabrication of high‐efficiency perovskite solar cells, yet heavily relied on multiple additives in upscalable fabrication in air. In this work, a simple α‐FAPbI_3_ ink is developed for the blade‐coating fabrication of phase‐pure α‐FAPbI_3_ in ambient air free from any additives containing extrinsic ions. The introduction of 2‐imidazolidinone (IMD) to the FAPbI_3_ precursor inks leads to the formation of intermediate phases that change the phase transition pathway from δ‐FAPbI_3_ to α‐FAPbI_3_ by tilting the PbI_6_ octahedrons with strong coordination to Pb^2+^. Furthermore, the IMD ligands in the intermediate phase gradually escape from the perovskite film during the annealing, leaving a phase‐pure α‐FAPbI_3_ film vertically grown with large grains. Consequently, the small‐sized PSCs fabricated with blade‐coated α‐FAPbI_3_ film achieve an efficiency of up to 23.14%, and the corresponding mini‐module yields an efficiency of 19.66%. The device performance is among the highest reported for phase‐pure α‐FAPbI_3_ PSCs fabricated in the air without non‐native cations or chloride additives, offering a simple and robust fabrication approach of phase‐pure α‐FAPbI_3_ films for PV application.

## Graphical abstract

With a stable 2‐imidazolidinone‐coordinated intermediate adduct regulating the growth of perovskite, pure α phase formamidinium lead iodide films are fabricated by blade‐coating in ambient air from a simple ink without any additives containing methylammonium caesium, bromide and chloride ions.

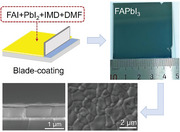

## 1 Introduction

Perovskite solar cells (PSCs) employing formamidine lead iodide (FAPbI_3_) have demonstrated exceptional potential in low‐cost photovoltaic (PV) applications over the last decade.^[^ ^1^, ^2^, ^3^ ^]^ Laboratory‐scale devices with areas less than 1 cm^2^ have attained power conversion efficiencies (PCEs) surpassing 26.7%.^[^ ^4^, ^5^ ^]^ Within the spectrum of lead halide perovskite materials, FAPbI_3_ has been considered the most highly efficient and widely used for single junction PSCs due to its low band gap (1.48 eV) and good thermal stability.^[^ ^1^, ^6^, ^7^, ^8^ ^]^ Nevertheless, at room temperature, α‐FAPbI_3_ is prone to spontaneously converting to the inactive δ phase, which is a process driven by the instability of the cubic lattice associated with the large ionic radius of FA^+^.^[^ ^9^ ^]^ Addressing this issue involves stabilizing α‐FAPbI_3_ through partial substitution of native ions with extrinsic ions such as methylammonium (MA^+^),^[^ ^10^ ^]^ cesium (Cs^+^),^[^ ^11^ ^]^ rubidium (Rb^+^)^[^ ^12^ ^]^ cations, or bromide (Br^−^)^[^ ^1^ ^]^ anions. Such alloying of multiple ion species in FAPbI_3_, however, can inadvertently lead to increased band gaps and compositional segregation.^[^ ^13^, ^14^ ^]^ In this regard, the addition of MACl, which can be largely removed during the post‐annealing, demonstrates its effectiveness in mitigating these issues and has become a predominant approach in preparing α‐FAPbI_3_. However, it is hard to remove the MA^+^ and Cl^−^ ions completely from the resulting film, which sets a potential risk for the long‐term stability of the device. Therefore, it is particularly important to explore the crystallization of phase‐pure α‐FAPbI_3_ without the addition of extrinsic ions.

Currently, most of the high‐performance PSCs are fabricated with perovskite film spin‐coated in laboratories, which can be hardly applied in upscale production. In contrast, the blade coating method, known for its high material efficiency and simplicity, has been extensively applied in the production of large‐area films.^[^ ^15^, ^16^, ^17^, ^18^ ^]^ However, in order to fabricate highly compact and uniform large‐area perovskite films by blade‐coating, multiple additives have been extensively added in the perovskite precursor solutions,^[^ ^19^, ^20^, ^21^ ^]^ which are also known as perovskite inks. Although yielding improved the quality of the mixed cation perovskite film, the phase purity of the perovskite is compromised, putting the device at risk of chemical segregation under long‐term operation. Therefore, there is a pressing need to develop perovskite inks with fewer additives for blade‐coating and other upscalable fabrication of phase‐pure α‐FAPbI_3_ films. Since the main solvent of DMF evaporates rapidly during annealing, DMSO and other Lewis base solvents have been employed to retard the crystalline growth as a ligand additive.^[^ ^22^, ^23^, ^24^ ^]^ However, the removal of the liquid additives usually leads to the formation of voids at the buried interface,^[^ ^25^ ^]^ which can only be mitigated by alloying with other non‐native ions.^[^ ^22^ ^]^ Therefore, it is imperative to explore solid Lewis bases as additives to regulate the nucleation and crystallization for upscalable fabrication of phase‐pure FAPbI_3_ films and devices.

Herein, we report a simple perovskite ink for blade‐coating fabrication of α‐FAPbI_3_ films in air, which is only composed of PbI_2_, FAI, DMF, and 2‐imidazolidinone (IMD) without any other extrinsic ions (MA^+^, Cs^+^, Rb^+^, Br^−^, Cl^−^, etc.). As a solid‐state Lewis base additive with strong coordination capability, IMD works effectively in modulating the crystallization of perovskite films by providing a new phase‐transition pathway in forming α‐FAPbI_3_. The addition of IMD also facilitates in‐air preparation of high‐quality α‐FAPbI_3_ films, yielding uniformly enlarged grains. The small area device and large area minimodules prepared with the blade‐coated film using the IMD incorporated inks demonstrate PCEs of 23.14% and 19.66%, respectively, demonstrating its potential for upscaling fabrication of α‐FAPbI_3_ films for commercial PV application.

## 2 Results and Discussion

### 2.1 Nucleation and Crystallization Processes of FAPbI_3_ Perovskite Films

**Figure 1:**
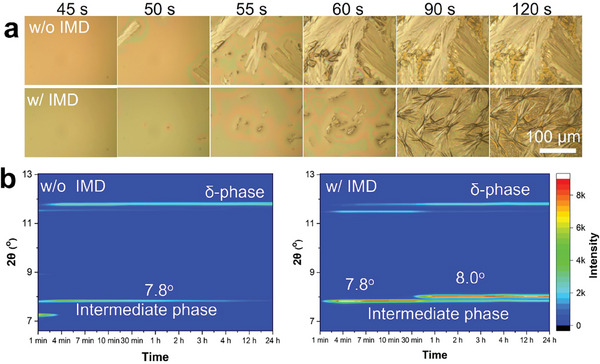
a) Optical microscope images of the natural drying and crystallization process of FAPbI_3_ precursor films without IMD (w/o IMD) and with IMD (w/ IMD); b) XRD evolution mapping of the films prepared from the FAPbI_3_ ink without IMD and with IMD.

To investigate the role of IMD additive in the nucleation and crystallization kinetics of the FAPbI_3_ precursor inks (FAI and PbI_2_ stoichiometrically dissolved in DMF), the natural drying process of the blade‐coated ink films with and without IMD is first compared. From the observation under an optical microscope (**Figure** **1**a), the blade‐coated ink films without IMD first form relatively small nuclei within the film through a random Poisson process. Subsequently, these nuclei undergo rapid 1D growth, leading to the development of dendrites that extend up to several hundred microns in length.^[^ ^26^ ^]^ After the wet film was completely dried, scanning electron microscopy (SEM) showed porous dendrites (Figure S1, Supporting Information). The observation is consistent with previous literature reports where only DMF solvent is being used.^[^ ^27^, ^28^ ^]^ From the X‐ray diffraction (XRD) evolution mapping shown in Figure 1b, we noted a gradual disappearance of the diffraction peak at 7.8° corresponding to the intermediate phase FA_2_Pb_3_I_8_·4DMF^[^ ^28^ ^]^ and a gradual appearance of a diffraction peak at 11.8° corresponding to the δ‐phase FAPbI_3_, indicating the removal of DMF in natural drying given sufficient time. In contrast, with the introduction of IMD, we found that the growth rate of dendrites under natural drying is dramatically suppressed (Figure 1a), presenting a similar role of MACl additive (Figure S2, Supporting Information). Besides, a large number of small hexagonal grains in between the dendrites are also observed, suggesting that the addition of IMD promotes uniform nucleation and that the grain growth process in this region aligns with the Avrami model.^[^ ^29^ ^]^ The colloidal nature of the perovskite precursor inks significantly affects the nucleation and growth of perovskite crystals.^[^ ^30^, ^31^ ^]^ The dynamic light scattering spectrum (DLS) of the precursor solution (Figure S3, Supporting Information) reveals that the addition of IMD to the ink impedes the aggregation of colloids and decreases their particle size. This effect sets a higher energy barrier for nucleation,^[^ ^32^, ^33^ ^]^ thereby explaining the delayed nucleation observed during the natural drying process. From the XRD evolution mapping shown in Figure 1b, the appearance of δ‐phase FAPbI_3_ is delayed. A new diffraction peak at 8.0° emerged with the fading of FA_2_Pb_3_I_8_·4DMF. The new diffraction peaks can be considered to be related to the IMD‐incorporated intermediate phase, which suppressed the release of DMF from the FA_2_Pb_3_I_8_·4DMF intermediate phase.

**Figure 2:**
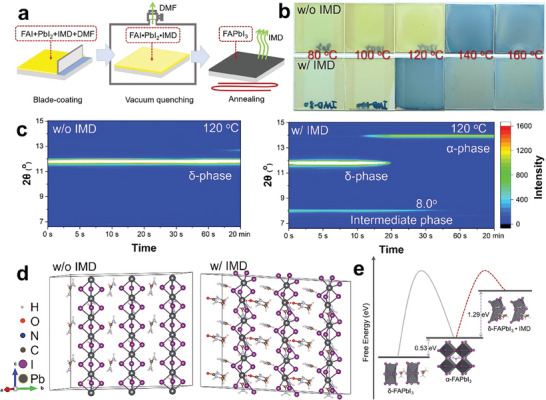
a) Schematic diagram of blade‐coating vacuum‐assisted preparation of FAPbI_3_ perovskite film; b) Photos of perovskite films without IMD and with IMD annealed at different temperatures; c) XRD evolution of the perovskite films without IMD and with IMD annealed at 120 °C; d) DFT calculations of δ‐phase FAPbI_3_ structure from side view without IMD and with IMD; e) Free‐energy calculation from δ‐phase to α‐phase FAPbI_3_ without IMD and with IMD.

To facilitate the rapid removal of excessive DMF from the blade‐coated wet film and accelerate the nucleation, a vacuum quenching treatment (**Figure** **2**a) is employed in obtaining a compact film without dendrites. During vacuum, the incorporation of IMD contributes to forming a denser film of the intermediate phase which improves the compactness of the perovskite film (Figures S4 and S5, Supporting Information). By varying the annealing temperatures on the blade‐coated films quenched with vacuum, it can be found that films without IMD turned black at 140 °C and above, indicating a successful transition from yellow δ‐FAPbI_3_ to black α‐FAPbI_3_. Interestingly, the addition of IMD effectively reduced the phase transition temperature to 120 °C (Figure 2b). The corresponding XRD patterns as shown in Figure S6 (Supporting Information) confirmed phase‐pure α‐FAPbI_3_ can be obtained by annealing at 120 °C with the assistance of IMD, suggesting that IMD may contribute to a lower temperature crystallization pathway to α‐FAPbI_3_. The evolution of XRD patterns as a function of annealing time (Figure 2c) showed that the vacuum quenched film without IMD kept δ‐FAPbI_3_ during annealing at 120 °C, confirming substantial removal of DMF by vacuum quenching process. Contrastingly, the film with the addition of IMD exhibits an additional diffraction peak of the IMD‐incorporated intermediate phase, which retains much longer than that of δ‐FAPbI_3_ before eventually converting to α‐FAPbI_3_. This transformational behavior mirrors previous findings where δ‐FAPbI_3_ initially vanished upon annealing samples with NMP + MACl^[^ ^34^ ^]^ or DMSO + MACl,^[^ ^35^ ^]^ followed by the intermediate phases fully converting to α‐FAPbI_3_ as annealing duration extended, suggesting that IMD might work similarly to MACl additive.

To reveal the influence of IMD on the phase transformation kinetics of FAPbI_3_, density functional theory (DFT) calculations were performed to optimize the δ‐FAPbI_3_ structures with and without IMD. Simulation results from Figure 2d and Figures S7 and S8 (Supporting Information) indicate that the IMD‐incorporated PbI_6_ framework undergoes significant tilting, with one bond length extending from 3.2 to 4.9 Å, and the opposite bond contracting to 3.1 Å, concurrent with a decrease in the bond angle from 75.9° to 62.4°. This structural alteration compromises the stability of the PbI_6_ framework, facilitating the disruption of the face‐sharing Pb‐I framework to yield a corner‐sharing configuration.^[^ ^3^, ^22^ ^]^ DFT calculations reveal that the energy difference between δ‐FAPbI_3_ and α‐FAPbI_3_ phases is 0.53 eV (Table S1, Supporting Information). In contrast, the energy difference between the IMD‐incorporated intermediate phase and α‐FAPbI_3_ is −1.29 eV. This indicates that the IMD‐incorporated intermediate phase requires less energy to transit into α‐FAPbI_3_, which explains the reduced α‐phase transition temperature compared to the film without incorporation of IMD.

It is worth noting that IMD has a lager dipole moment compared to the conventionally used ligand solvent of DMSO and NMP (Figure S9, Supporting Information), demonstrating its capacity to form a more stable intermediate phase with FAPbI_3_.^[^ ^16^, ^36^ ^]^ This can be further evidenced by stable XRD patterns of the intermediate films (vacuum‐quenched film before annealing) during aging in ambient air (Figure S10, Supporting Information). The IMD film aged for 380 h is still capable of obtaining high‐quality α‐FAPbI_3_ films after annealing, whereas the control film with DMSO and NMP has showed reduced crystallinity and significant precipitation of PbI_2_. Although IMD is in a solid state at room temperature with higher melting points and lower vapor pressures than DMSO and NMP, the coordinated IMD can be escaped from the intermediate phase under its boiling temperature through sublimation (Figures S11 and S12, Supporting Information). According to the thermogravimetric analysis (TGA) of the wet film (blade‐coated without annealing) powders prepared with IMD, notable weight loss at 92 °C is observed on IMD‐incorporated film powders, whereas the FAPbI_3_ films start losing weight above 250 °C (Figure S13, Supporting Information). To reveal if IMD can be fully removed from the film by annealing at c, the X‐ray photoelectron spectra (XPS) of the films prepared with and without IMD are compared (Figure S14, Supporting Information). With the addition of IMD, the C1s and N1s XPS peaks correspond to FA cations^[^ ^37^ ^]^ in the blade‐coated film before the annealing shift toward lower binding energy. However, the shifts are restored when the film is annealed, which indicates the influence of IMD on the FA cations in the film is eliminated by thermal annealing. The interaction between IMD and FA cations can be further evidenced by Fourier transform infrared (FTIR) spectroscopy (Figure S15, Supporting Information). After annealing, the stretching vibration peak of C─N from IMD is not detected, and the blueshifted stretching vibration peak of C═N from FA cations^[^ ^24^ ^]^ also restores to its original position at 1705 cm^−1^. These results collectively demonstrate the IMD additive can be completely removed upon thermal annealing at 120 °C.

### 2.2 Optimization of Films

**Figure 3:**
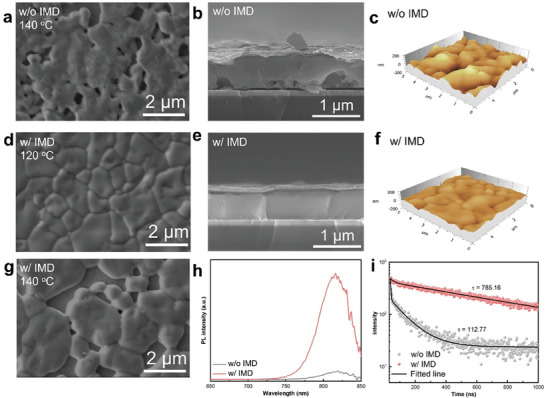
Top‐view SEM images of the perovskite films a) without IMD (140 °C), with IMD at d) 120 °C and g) 140 °C; Cross‐sectional SEM images of the perovskite films b) without IMD and e) with IMD; AFM spectrum of the perovskite films c) without IMD and f) with IMD; h) PL spectrum of the perovskite films without IMD and with IMD, i) TRPL spectrum of the perovskite films without IMD and with IMD.

SEM imaging of perovskite films annealed at different temperatures (**Figure** **3**a; Figure S16, Supporting Information) showed that samples without IMD exhibited poor surface coverage at all annealing temperatures. In contrast, the phase‐pure α‐FAPbI_3_ obtained with IMD at 120 °C exhibited a compact polycrystalline morphology (Figure 3d), which is promising for making highly efficient perovskite solar cells. However, at higher temperatures, the films developed significant voids as the grains grew lager (Figure 3g; Figure S16, Supporting Information). This could be related to the incomplete grain growth of the intermediate phase due to its rapid deconstruction under 140 °C, which impedes the fine transformation from the intermediate phase to α‐FAPbI_3_ (Figure S17, Supporting Information). It is worth noting that the film without IMD turned black upon 140 °C annealing for 60 s, whereas the film with IMD only required 20 s (Figure S18, Supporting Information). This further demonstrates the incorporation of IMD effectively promotes the phase transition to α‐FAPbI_3_. SEM images (Figure S19, Supporting Information) of perovskite films at different annealing times reveal that films without IMD consistently exhibited poor surface coverage, and contrastingly, films with IMD exhibited decent coverage with clear grains and displayed continuous grain growth over time. Considering the above result, it is inferred that the fabrication of dense perovskite films by blade‐coating requires 1) acquiring a sufficient quantity of nucleation sites during the nucleation phase, and 2) attaining an optimal annealing temperature conducive to phase transition, coupled with adequate time for the growth of crystal nuclei. At the given annealing temperature of 120 °C, a lower amount of IMD led to pinholes and a higher amount of IMD led to non‐uniform enlarged grains (Figure S20, Supporting Information), whereas 60% molar ratio of IMD:Pb is found to optimal in obtaining a decent film morphology, leading to optimized device performance (Figure S21, Supporting Information). Notably, this is very close to the molar ratio (50%) of IMD:Pb in the simulated δ‐FAPbI_3_:IMD intermediate structure. Therefore, a 60% molar ratio of IMD with 120 °C annealing temperature has been used in the fabrication of perovskite solar cells with the IMD‐added ink.

The cross‐sectional SEM image of α‐FAPbI_3_ films prepared without IMD displays significant pores, especially enriched at the buried interface. While films with IMD exhibit vertically grown crystals in large size (Figure 3b,e), demonstrating the effect of IMD in modulating crystallographic growth. In addition, atomic force microscopy (AFM) results show that the root mean square roughness of the perovskite film decreases from 57.0 to 19.3 nm with the addition of IMD (Figure 3c,f), which will improve the contact between the perovskite film and the electron transport layer. Carrier dynamics of films were studied using photoluminescence (PL) spectroscopy and time‐resolved photoluminescence (TRPL) measurements. The blade‐coated FAPbI_3_ films prepared with IMD exhibit an optical band gap of 1.52 eV (Figure S22, Supporting Information) with dramatically intense PL emission compared to that without IMD (Figure 3h), indicating that IMD‐associated delayed nucleation and assisted growth contribute to a dense perovskite film which effectively suppressed non‐radiative recombination. Meanwhile, the prolonged carrier lifetimes of the film prepared with IMD (Figure 3i) suggest fewer defects and notably reduced nonradiative recombination in these films as well, indicating a high‐quality crystallization of the blade‐coated FAPbI_3_ films can be achieved with the assistance of IMD.

### 2.3 Device Performance

**Figure 4:**
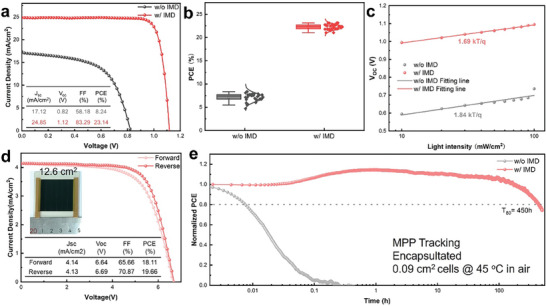
a) *J–V* spectrum without and with IMD: b) PCE spectrum without IMD and with IMD; c) Light intensity dependence without and with IMD; d) *J–V* of unencapsulated modules with IMD; e) Maximum power point tracking of cells in an ambient atmosphere (45 °C).

To demonstrate the performance of perovskite solar cells (PSCs) fabricated with the blade‐coated FAPbI_3_ films, the current density–voltage (*J–V*) characteristics of small‐area PSCs structured with ITO/(4‐(2,7‐dibromo‐9,9‐dimethylacridin‐10(9H)‐yl)butyl)phosphonic acid (DMAcPA)/Al_2_O_3_/PEABr/FAPbI_3_/PEABr/C_60_/SnO_2_/Ag^[^ ^38^ ^]^ were examined in the reverse scan (RS), as displayed in **Figure** **4**a. The device prepared without IMD exhibited a low power conversion efficiency (PCE) of 8.24%, which can be ascribed to the extensive porosity in the FAPbI_3_ film. The performance of the device is significantly enhanced with the addition of IMD, achieving a PCE of 23.14%, a short‐circuit current density (*J_SC_*) of 24.85 mA cm^−2^, an open‐circuit voltage (*V_OC_*) of 1.12 V, and a fill factor (FF) of 83.29%, with negligible hysteresis (Figure S23, Supporting Information). The external quantum efficiency (EQE) spectrum recorded an integrated photocurrent of 24.13 mA cm^−2^, in close agreement with the *J–V* measurements (Figure S24, Supporting Information). To demonstrate the reproducibility across devices, statistics from 30 cells were collected for both films with and without IMD as shown in Figure 4b, showing the narrow performance distribution of IMD devices. Additional tests assessing the dependence on light intensity were conducted on devices with and without IMD (Figure 4c). The result revealed that devices with IMD have an ideality factor of 1.69, which more closely approaches unity compared to the device without using IMD. This suggests a higher proportion of radiative recombination in devices with IMD and implies a reduction in non‐radiative recombination.

To demonstrate the capability of the IMD‐incorporated ink for upscaling application, 5 different spots on the 5×5 cm^2^ FAPbI_3_ film prepared by blade‐coating are examined with XRD and SEM. The results demonstrate remarkable uniformity in both crystallinity and surface morphology (Figure S25, Supporting Information). Subsequently, mini‐modules with 12.6 cm^2^ aperture area (geometric fill factor is 0.985, Figure S26, Supporting Information) are fabricated with the same p‐i‐n device structure to small area cells, positioning it amongst the most efficient FAPbI_3_ PSCs without the addition of MA^+^, Cs^+^, Rb^+^, Br^−^ or Cl^−^ containing additives (Table S2, Supporting Information). Furthermore, the blade‐coated α‐FAPbI_3_ perovskite film with IMD exhibits stable phase stability by maintaining its α‐phase structure for 744 h in the ambient atmosphere (temperature 15–20 °C, relative humidity 40–60%), while the film without IMD transitioned to the δ‐phase at 336 h and completely shifted to the δ‐phase after 44 additional hours (Figure S27, Supporting Information). Meanwhile, the blade‐coated α‐FAPbI_3_ perovskite film with IMD also exhibits decent thermal stability under continuous heating at 85 °C and kept its α‐phase structure for over 200 h (Figure S28, Supporting Information), which can be ascribed to its modulated crystallization under the assistance of IMD. Consequently, the IMD device demonstrates much superior operational stability compared to the control device (Figure 4e), maintaining 80% of its initial PCE after maximum power point (MPP) tracking for 450 h under continuous illumination (≈45 °C).

## 3 Conclusion

In summary, a simple blade‐coating ink free from any extrinsic ions is developed for the blade‐coating fabrication of α‐FAPbI_3_ films in ambient air. The incorporation of a solid‐state additive reduces the average size of colloidal in the perovskite inks, providing a fine nucleation environment. The tilted PbI_6_ framework with IMD incorporation facilitates the crystallization of α‐FAPbI_3_ at lower temperatures. After regulating the crystalline growth, the IMD molecules can be completely removed with thermal annealing, guaranteeing a phase‐pure FAPbI_3_ composition. The IMD added ink promotes the in‐air blade‐coated α‐FAPbI_3_ film yielding vertically grown large grains with a smooth and compact morphology, which demonstrates significantly improved optoelectronic properties. As a result, the small‐area PSCs obtained a PCE of 23.14% and the mini‐modules demonstrate a PCE of 19.66% with an aperture area of 12.6 cm^2^, which are among the highest for PSCs fabricated with blade‐coated perovskite free from MA^+^, Cs^+^, Br^−^, and Cl^−^ in air, offering a facile and reproducible approach for the scalable fabrication of pure‐phase FAPbI_3_ and PV devices in ambient air.

## Conflict of Interest

The authors declare no conflict of interest.

## Supporting information



Supporting Information

Supplemental Video 1

Supplemental Video 2

Supplemental Video 3

Supporting Information

## Data Availability

The data that support the findings of this study are available in the supplementary material of this article.
