# Supplementary material for: Methylammonium‐Free Ink for Blade‐Coating of Pure‐Phase α‐FAPbI3 Perovskite Films in Air
Source: Adv Sci (Weinh). 2024 Oct 22;11(46):2410266. doi: 10.1002/advs.202410266 (PMC11633507; doi:10.1002/advs.202410266)
Supplement: Supplementary file 1 — Supporting Information [file ADVS-11-2410266-s001.docx]

**Supporting Information**

**Methylammonium-Free Ink for Blade-Coating of Pure-Phase α-FAPbI_3_ Perovskite Films in Air**

Jianbo Liu^a^, Jingwen Cao^b^, Meng Zhang^b^*, Xiaoran Sun^a^, Tian Hou^a^, Xiangyu Yang ^a^, Linhu Xiang^a^, Xin Liu^a^, Zhipeng Fu^a^, Yuelong Huang^a,c^*, Feng Wang^d^, Wenhua Zhang^e^*, Xiaojing Hao^b^*

^a^School of New Energy and Materials, Southwest Petroleum University, Chengdu, 610500, China

^b^The Australian Centre for Advanced Photovoltaics, School of Photovoltaic and Renewable Energy Engineering, University of New South Wales, Sydney, New South Wales 2052, Australia

^c^Phoenixolar Optoelectronics Co., Ltd., Huzhou, Zhejiang, China

^d^Center for Combustion Energy, Department of Energy and Power Engineering, Tsinghua University, 100084 Beijing, China

^e^Yunnan Key Laboratory of Carbon Neutrality and Green Low-carbon Technologies, Yunnan Key Laboratory for Micro/Nano Materials & Technology, Southwest United Graduate School, School of Materials and Energy, Yunnan University, Kunming 650504, China

*Corresponding Authors. Email: meng.zhang@unsw.edu.au; hyl@phoenixolar.com; wenhuazhang@ynu.edu.cn; xj.hao@unsw.edu.au

1. **Experimental Section**

**Materials.**

Dimethylformamide (DMF), isopropanol (IPA), dimethyl sulfoxide (DMSO), 1-methyl-2-pyrrolidinone (NMP), and aluminum oxide (Al_2_O_3_) were procured from Sigma Aldrich. (4-(2,7-dibromo-9,9-dimethylacridin-10(9H)-yl)butyl)phosphonic acid (DMAcPA) with a purity of 99% was acquired from Borun New Material Technology Ltd. Phenylethylammonium bromide (PEABr) with a purity of 99.9% was sourced from TCI America. Formamidinium iodide (FAI) with a purity exceeding 99.5% was procured from Greatcell Solar Materials Pty Ltd. Lead (II) iodide (PbI_2_), surpassing a purity of 99.999%, was obtained from Xi’an Elante New Material Co., Ltd. 2-Imidazolidinone (IMD) with a purity greater than 97.0% was purchased from Aladdin. Fullerene (C_60_) was acquired from Xi’an Yuri Solar Co., Ltd. All chemicals were utilized as received without subsequent purification.

**Preparation of FAPbI3 perovskite precursor solution.**

A mixture containing 1.5 M FAI, 1.5 M PbI_2_ and IMD was prepared in 1 mL of DMF solvent. The resultant perovskite precursor solution was agitated for 30 minutes to ensure complete dissolution.

**Solar cell and minimodules fabrication.**

The ITO conductive glass substrate was ultrasonically cleaned with deionized water (containing 5% detergent by volume), deionized water, and ethanol for 20 minutes respectively, then dried with dry air and treated with UV ozone for 15 minutes. DMA-CPA (1 mg/mL in DMF) was deposited on ITO substrate at 4000 rpm for 30 s, and then annealed at 100 ^o^C for 30 minutes. And Al_2_O_3_ (the dilution volume ratio of the original solution and IPA is 1:50) dispersion solution was spin-coated on the DMAcPA film at 5000 rpm for 30 s and heated at 100 ^o^C for 10 min. Then, the substrate was treated with PEABr solution (1 mg/mL in DMF) through spin coating at 5000 rpm for 30 s and subsequently kept at 100 ^o^C for 5 min on a hot plate. The process of preparing a perovskite film in air is as follows: drop the perovskite precursor solution (usually 15 µL for a 2.5×2.5 cm^2^ substrate and 30 µL for a 5×5 cm^2^ substrate) into the gap between the scraper and the substrate (~150 µm) and then blade coating at a speed of 5 mm/s. Then the wet film is quickly transferred to the vacuum chamber for a vacuum flash evaporation process. The vacuum flash evaporation time is pumped to about 5 Pa within 20 seconds, and then placed on a hot stage for annealing at different temperatures for 20 minutes. After preparation of the perovskite layer, PEABr solution (1 mg/mL in IPA) was spin-coated on the top of perovskite film at 5000 rpm for 30 s, followed by keeping at a hot plate for 10 min with 100 ^o^C. The 20 nm C_60_ was successively thermally evaporated with an evaporation rate of 1.0 Å/s at a pressure of around 1*10^-3^ Pa. Thereafter, 20 nm of SnO_2_ was deposited on top of the C_60_ substrate by 75 cycles in an ALD reactor at 100 °C. Each ALD cycle consisted of 8 s of TDMA dose, followed by 18 s of purge, then 6 s of water vapor dose, followed by 18 s of purge. Finally, the device is completed by thermal evaporation of silver (120 nm) under vacuum.

Module Fabrication: To fabricate perovskite solar modules, the layers consisting of ITO/hole transport layer (HTL)/FAPbI_3_/electron transport layer (ETL)/Ag electrodes are sequentially laser-scribed to delineate a P1-P2-P3-P4 pattern. The delineation results in active areas measuring 0.093 cm^2^ for the smaller device and 12.6 cm^2^ for the larger module.

**Characterization of devices and films.**

The X-ray diffraction (XRD) analysis of the film series was conducted using a Dandong Tongda Science and Technology Co., Ltd. X-ray diffractometer, employing Cu Kα radiation (λ = 1.5418 Å). The films were examined via top-view scanning electron microscopy (SEM) employing a Beijing KYKY Optic-electronics Technology Co., LTD., KYKY-EM8000 microscope. Ultraviolet-visible (UV-vis) absorption spectra were recorded using a Shanghai Metash Instruments Co., Ltd., Q6 spectrometer. Thermogravimetric analysis (TGA) spectra were acquired with a Rigaku TG/DTA8122 thermogravimetric analyzer at a nitrogen flow rate of 20 mL/min and a temperature increase rate of 10 °C/min from 25 °C to 400 °C. Dynamic light scattering (DLS) experiments were conducted using a Malvern Zetasizer Nano ZS90. X-ray photoelectron spectroscopy (XPS) analyses of the film series were performed with a Thermo Scientific K-Alpha spectrometer. Steady-state photoluminescence (PL) and time-resolved photoluminescence (TRPL) measurements were obtained using an Edinburgh Instruments FLS 980 PL spectrometer, with excitation sources of 468 nm continuous wave and 406 nm pulsed laser, respectively. Fourier-transform infrared (FTIR) spectra were recorded using BRAIC, WQF-520 FTIR spectrometers. The current-voltage (J-V) characteristics of the perovskite solar cells were assessed using a Keithley B2901A source meter under simulated AM 1.5 G illumination (100 mW/cm^2^) provided by an Enli Tech solar simulator. The external quantum efficiency (EQE) spectrum was determined employing a BENTHAM TMC300 EQE system, with a scanning range of 300-900 nm. Atomic force microscopy (AFM) images were acquired using a KEYSIGHT Technologies 7500 AFM.

**DFT calculation.**

The first-principles density functional theory (DFT) simulations were implemented with the Vienna Ab Initio Simulation Package (VASP)^[1, 2]^ to study the geometric structures and energies of the δ-FAPbI3 and δ-FAPbI3-IMD perovskite. The generalized gradient approximation exchange-correlation functional of Perdew–Burke–Ernzerhof (PBE) was adopted in the calculations within the generalized gradient approximation (GGA)^[3]^. The energy cutoff for the plane wave basis set was 520 eV. During the optimization of the geometries, to ensure that each atom was in mechanical equilibrium, all structures were allowed to keep relax until both the norms of all the forces were smaller than 0.02 eV Å−1 and total energy changes were smaller than 1×10-6 eV. All calculation illustrations were performed using VESTA^[4]^.


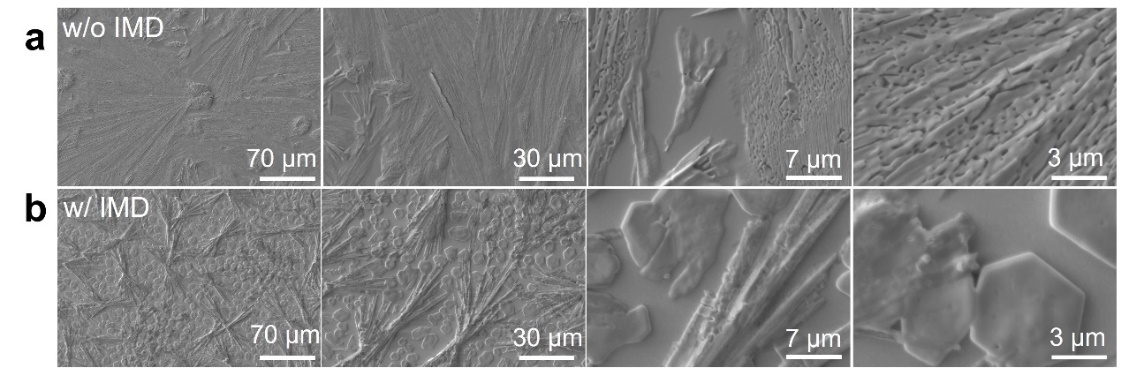


Figure S1. SEM of perovskite films (a) without IMD and (b) with IMD after natural drying at different magnifications.


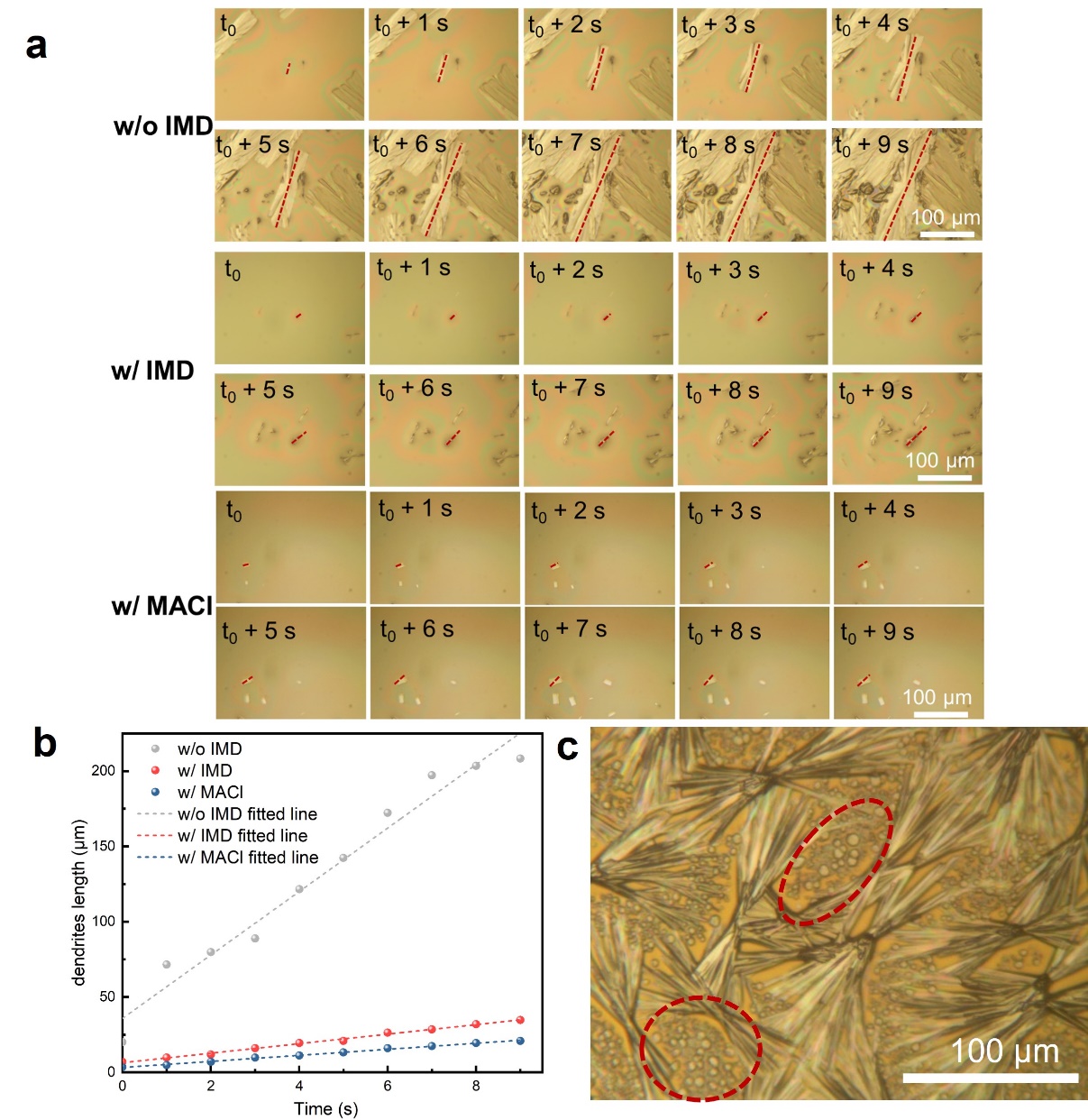


Figure S2. (a) The dendrite growth during natural drying of FAPbI_3_ precursor films without IMD, with IMD and with MACl. (b) Line graph of dendrite length change over time. (c) Microscope photo of FAPbI_3_ precursor film with IMD after natural drying.


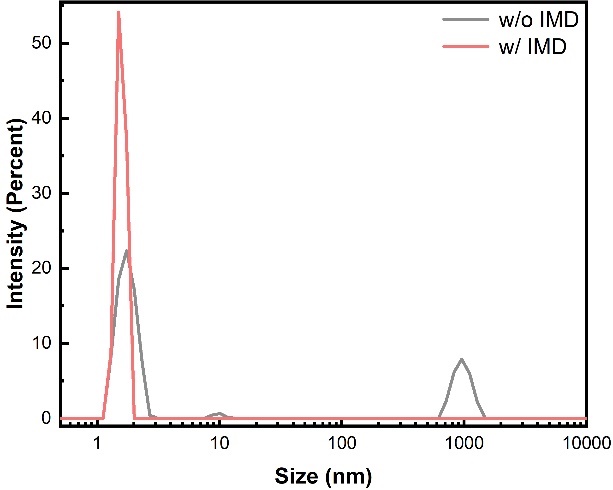


Figure S3. DLS pattern of without IMD and with IMD perovskite precursor solution, the concentration of the test sample is 1.5 M.


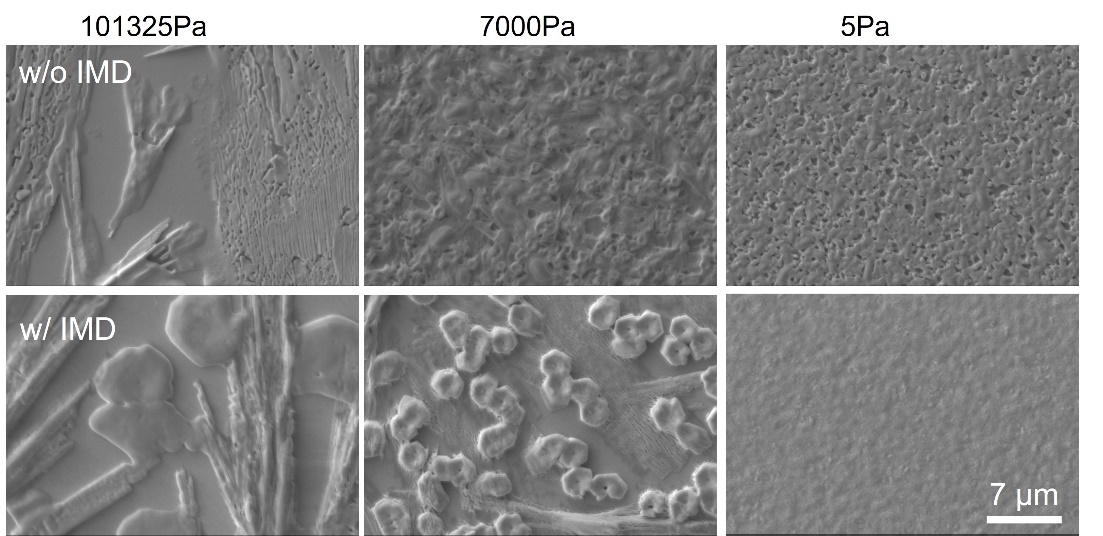


Figure S4. SEM images of perovskite films without and with IMD quenched at different vacuum levels.


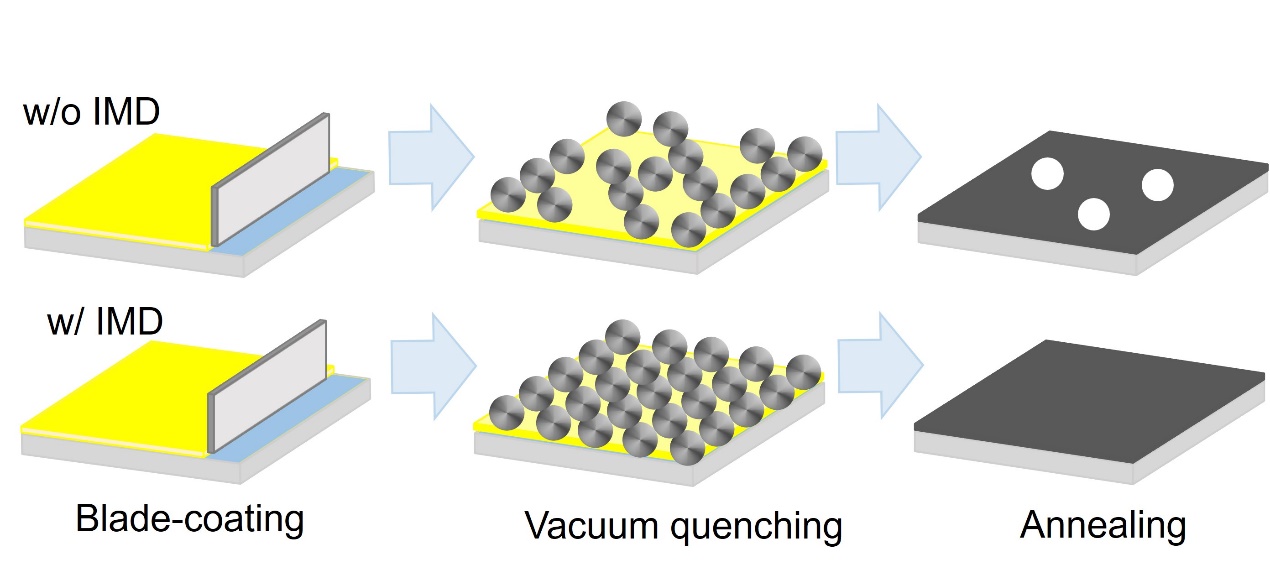


Figure S5. Schematic illustration of the perovskite films fabrication without and with IMD by blade-coating in air.


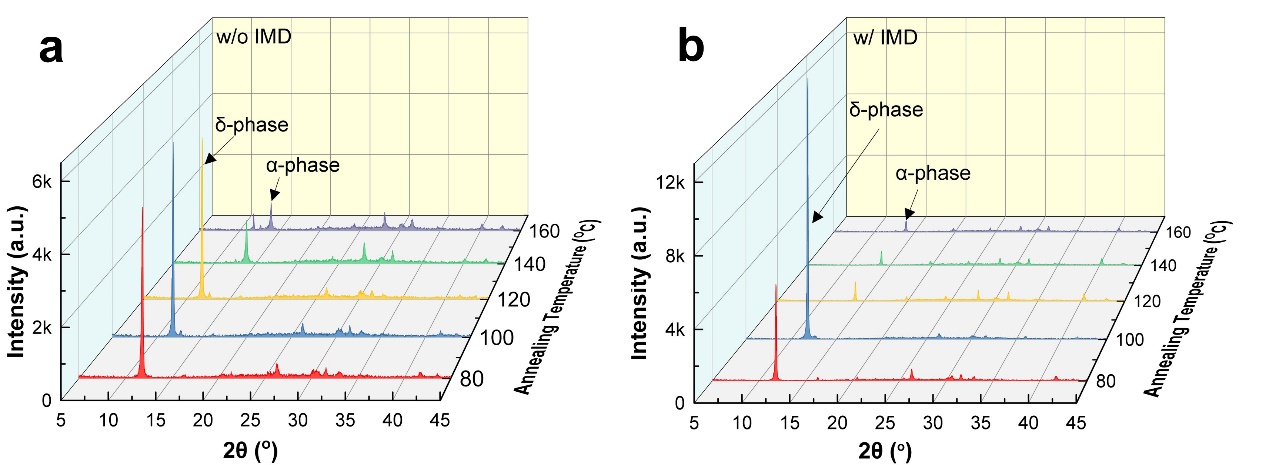


Figure S6. XRD patterns of the perovskite films (a) without IMD and (b) with IMD at different annealing temperatures.


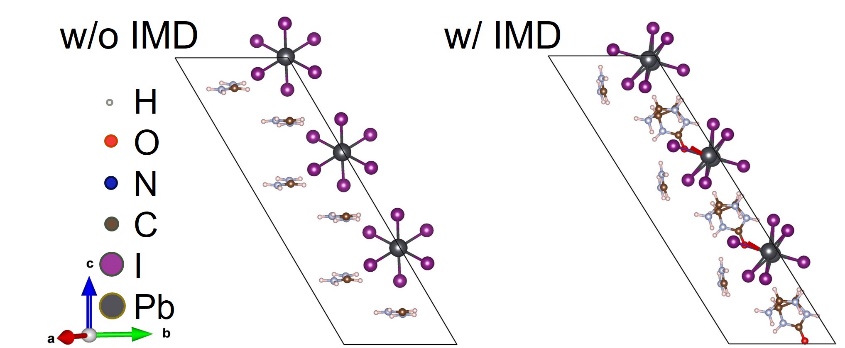


Figure S7. DFT calculations of δ-FAPbI_3_ structure from top view without and with IMD.


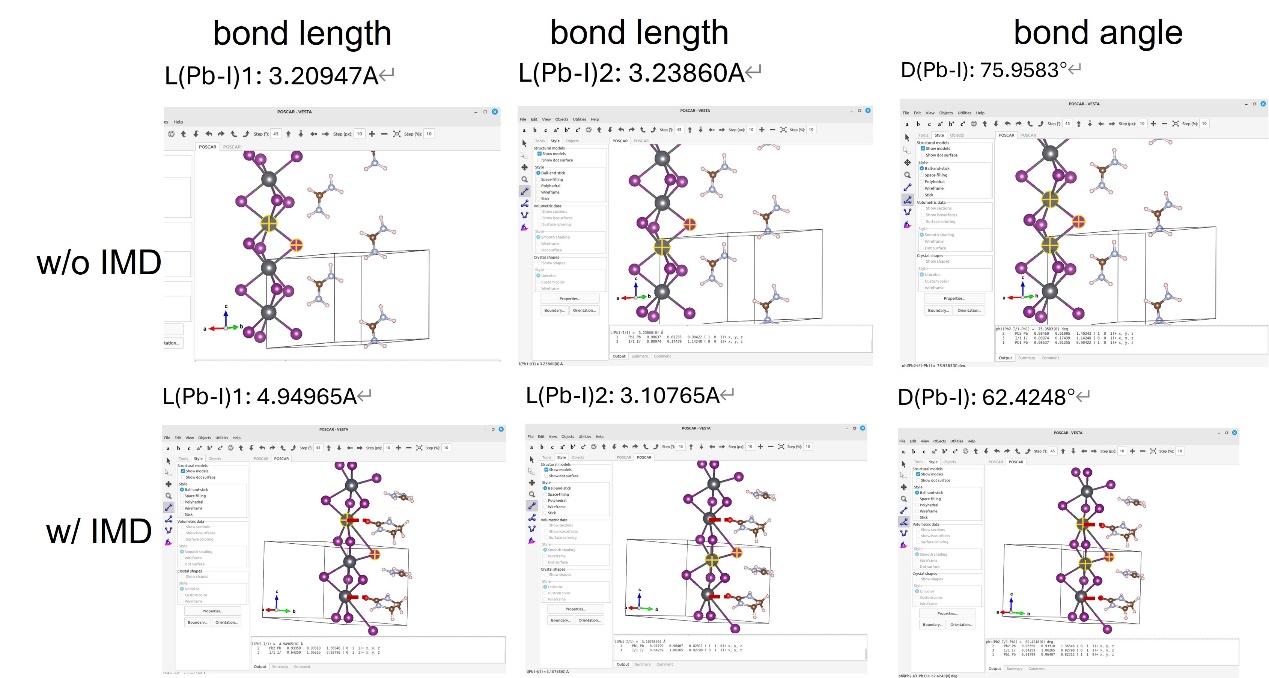


Figure S8. Bond length and bond angle of PbI_6_ framework without and with IMD.


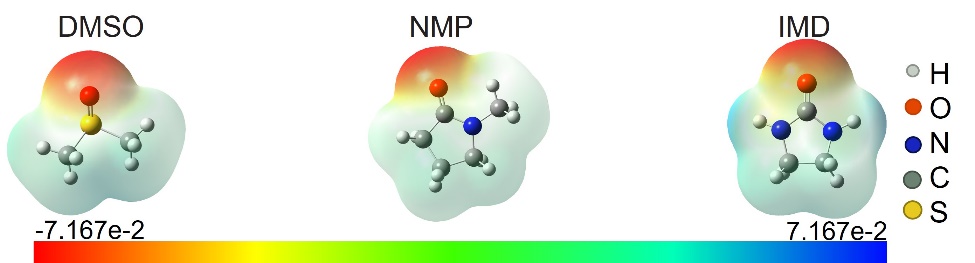


| Additive | DMSO | NMP | IMD |
| --- | --- | --- | --- |
| electric dipole (Debye) | 4.50 | 4.09 | 4.58 |

Figure S9. Electrostatic potential diagram and electric dipole of DMSO, NMP and IMD.


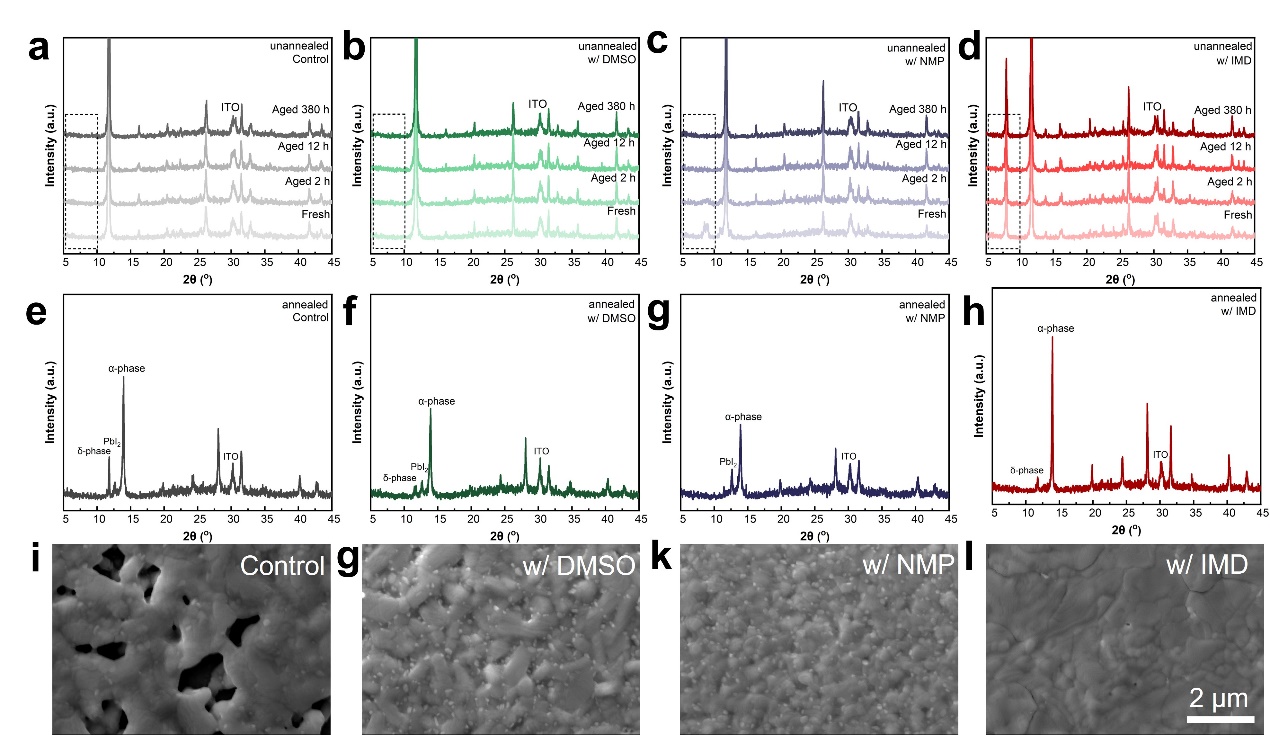


Figure S10. XRD patterns of unannealed intermediate films aged at different times (a) control, (b) w/ DMSO, (c) w/ NMP, and (d) w/ IMD; XRD patterns of 380-hours-aged intermediate films after annealing (e) control, (f) w/ DMSO, (g) w/ NMP, and (h) w/ IMD; SEM images of 380-hours-aged intermediate films after annealing (i) control, (g) w/ DMSO, (k) w/ NMP, and (l) w/ IMD.


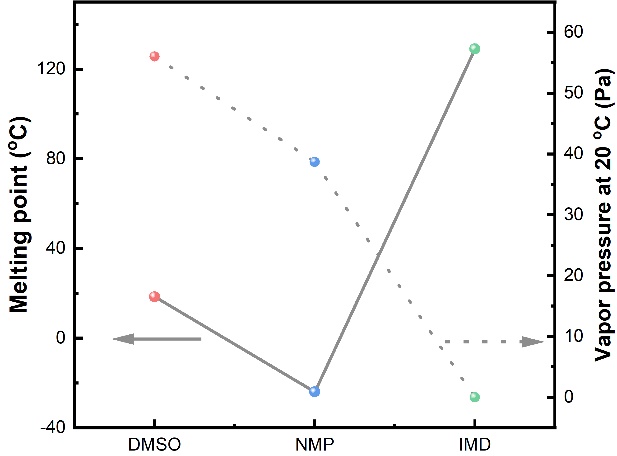


Figure S11. Melting point and vapor pressure at 20 ^o^C of DMSO, NMP and IMD.


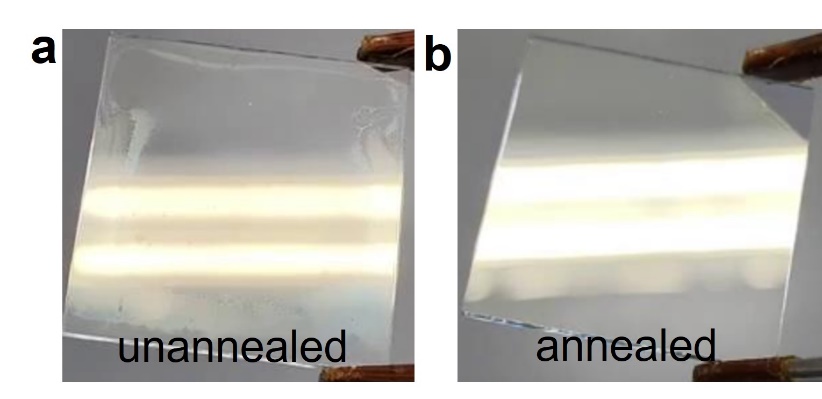


Figure S12. Photos of (a) unannealed and (b) annealed ITO glass substrates containing IMD.

When a solution containing IMD is scraped onto an ITO glass substrate, it can be seen that solid matter exists on the glass surface. After annealing at 120 ^o^C, the glass surface becomes smooth. Therefore, it can be concluded that the IMD is removed in the form of sublimation during annealing.


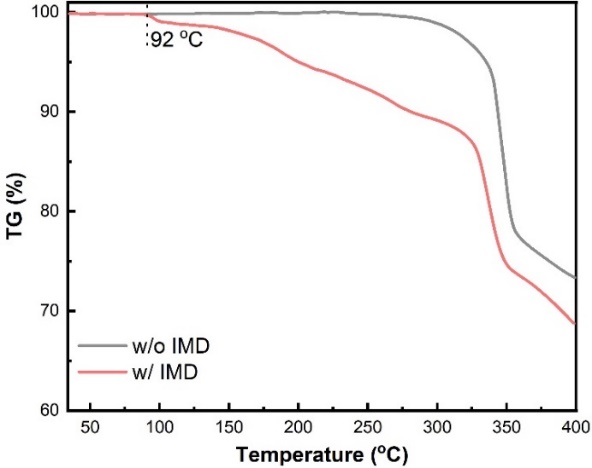


Figure S13. Thermogravimetric analysis of without and with IMD perovskite powder.


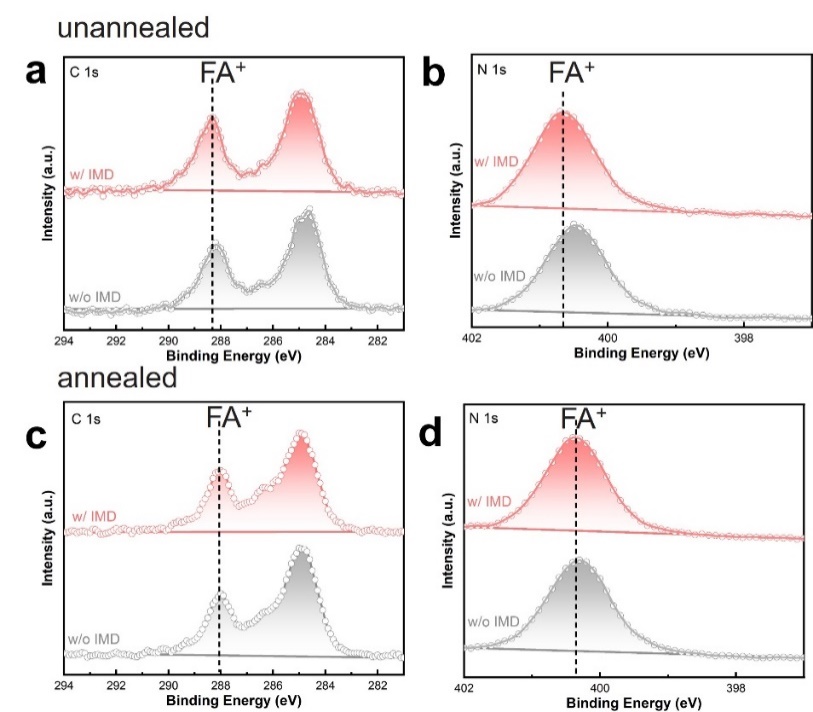


Figure S14. XPS pattern of unannealed and annealed the perovskite films without and with IMD.


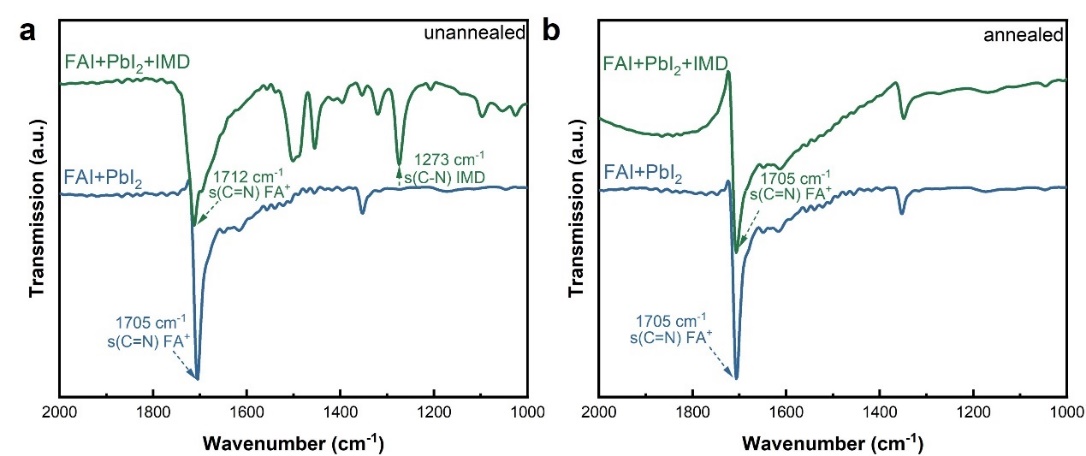


Figure S15. (a) unannealed and (b) annealed FTIR patterns FAPbI_3_ films without and with IMD.


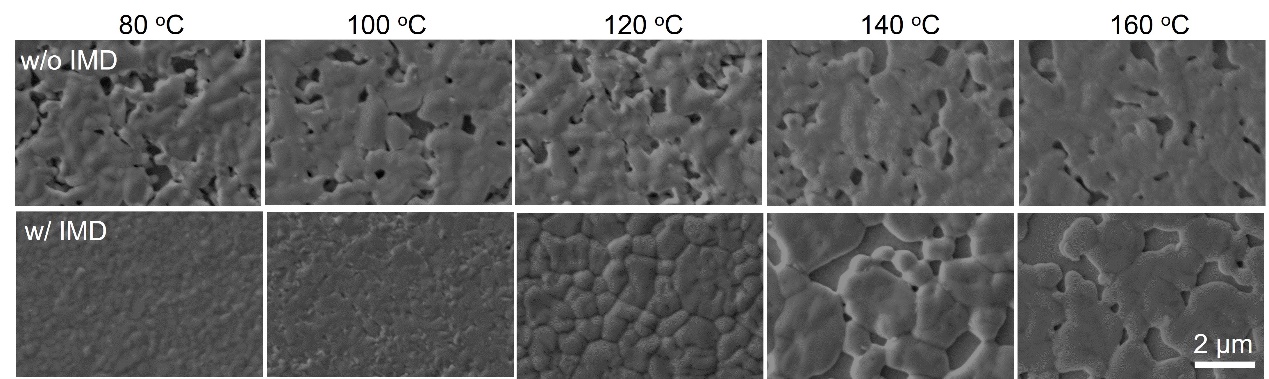


Figure S16. Top-view SEM images of the perovskite films (c) without IMD and (d) with IMD at different annealing temperatures (scale bars: 2 µm).


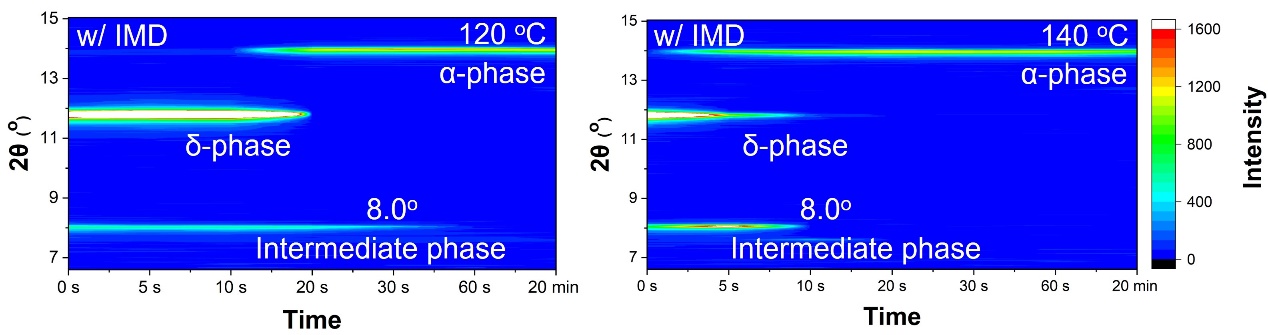


Figure S17. XRD evolution of perovskite films prepared with IMD under 120 °C and 140 °C.


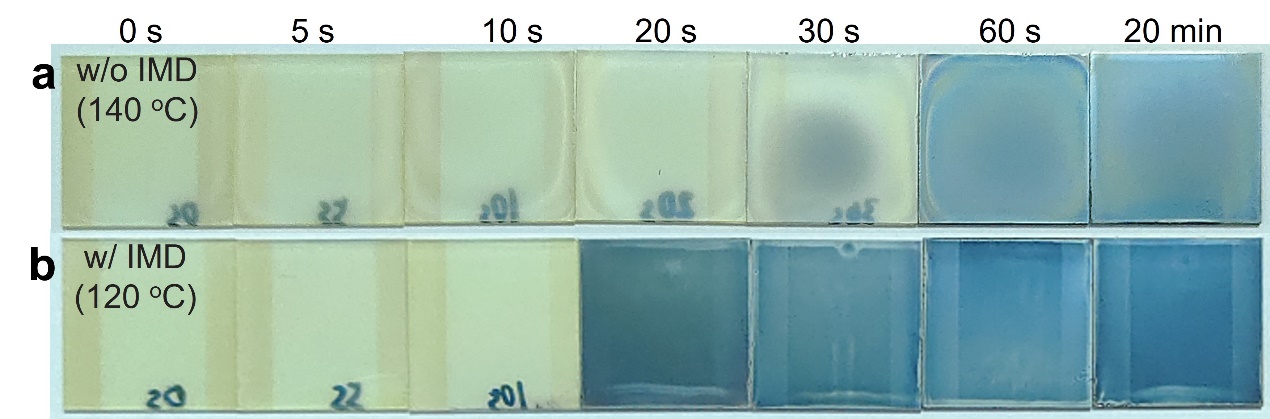


Figure S18. Photos of the FAPbI_3_ perovskite films (c) without IMD and (d) with IMD for different annealing times.


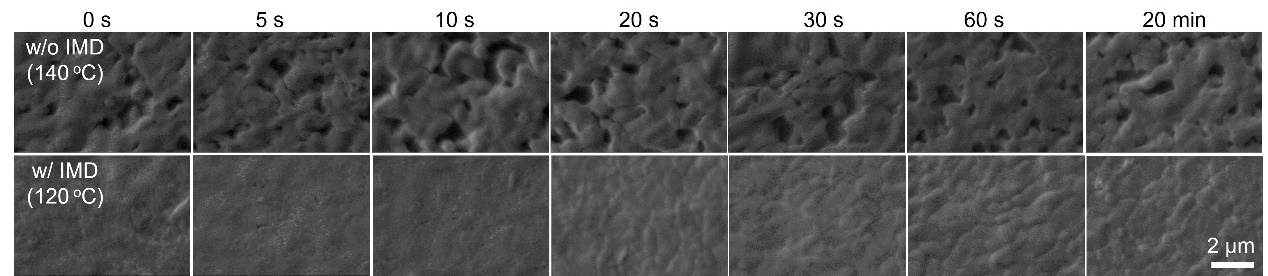


Figure S19. SEM of the FAPbI_3_ perovskite films (c) without IMD and (d) with IMD for different annealing times.


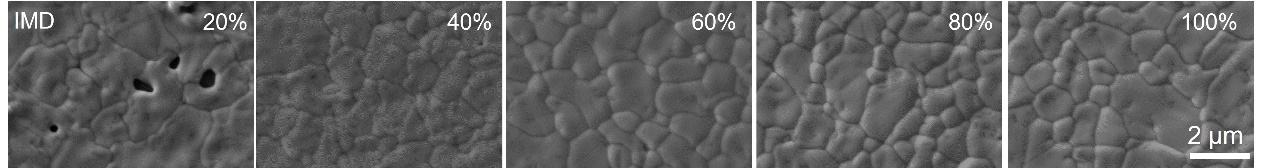


Figure S20. SEM of FAPbI_3_ with different molar amounts of IMD.


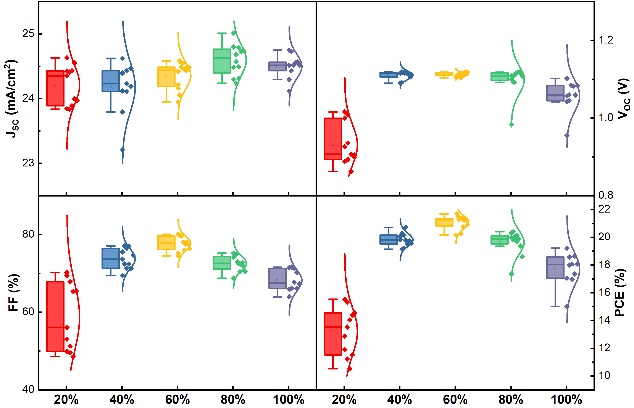


Figure S21. PV parameters distributions of the devices prepared with different molar ratio of IMD:Pb.


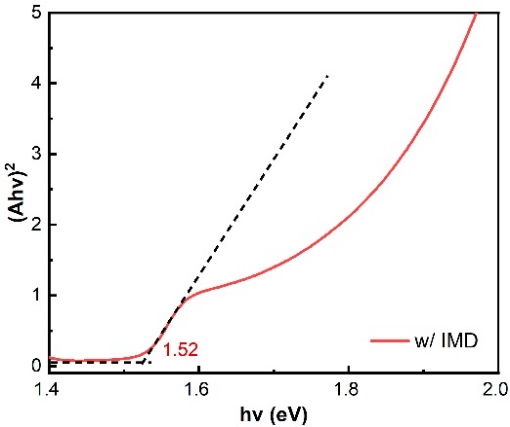


Figure S22. Tauc plot of the blade-coated α-FAPbI_3_ perovskite films.


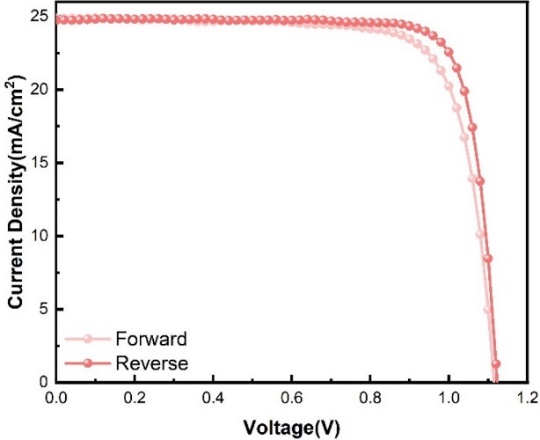


|  | J_sc_  (mA/cm^2^) | V_oc_  (V) | FF  (%) | PCE  (%) |
| --- | --- | --- | --- | --- |
| Forward | 24.87 | 1.11 | 76.88 | 21.33 |
| Reverse | 24.73 | 1.12 | 82.00 | 22.77 |

Figure S23. The *J-V* curves of forward and backward scanning for phase-pure α-FAPbI_3_ PSC.


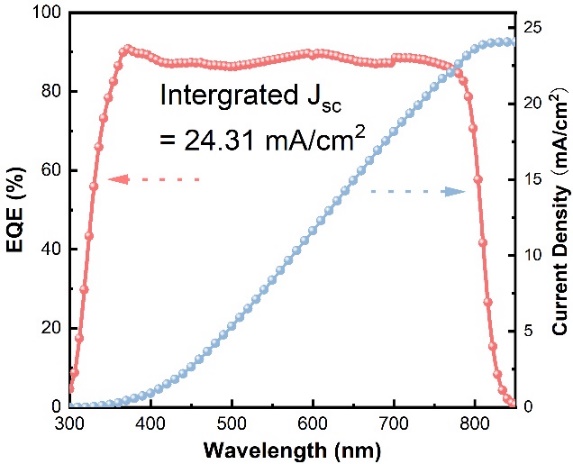


Figure S24. EQE spectrum of FAPbI_3_ device fabricated with IMD


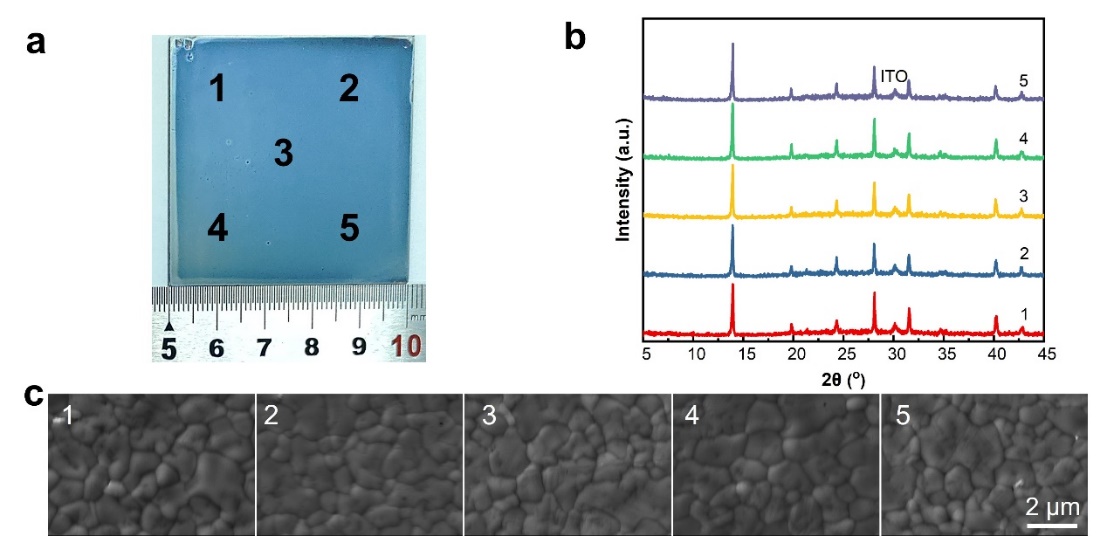


Figure S25. (a) Photograph of a 5×5 cm^2^ FAPbI_3_ film prepared by blade-coating with the IMD ink. (b) XRD patterns and (c) SEM images collected at 5 different spots on the film.


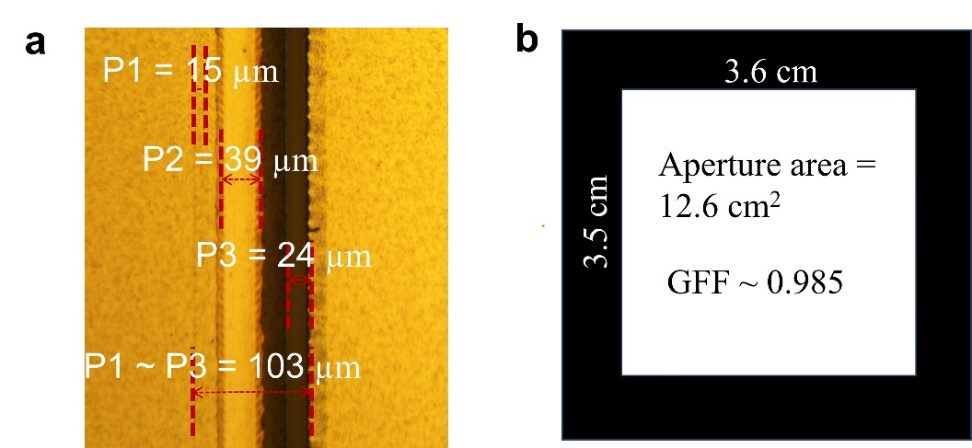


Figure S26. (a) The optical image of the P1-P2-P3 patterning of the mini-module. (b) Aperture size illustration.


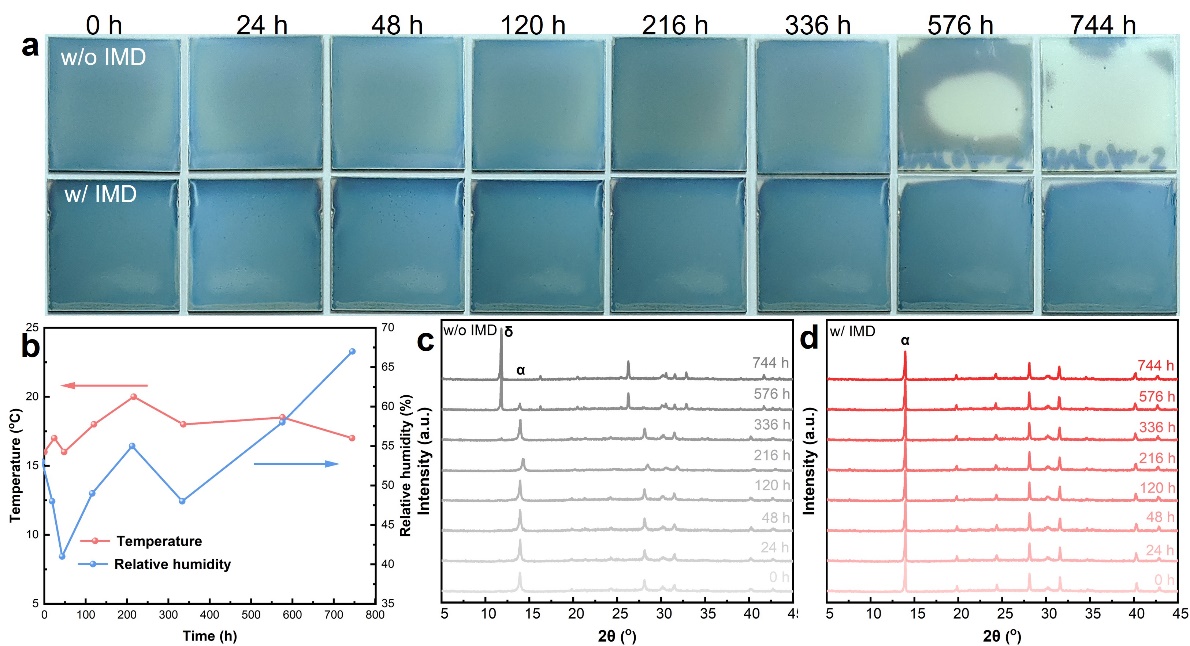


Figure S27. Moisture stability in the environment of FAPbI_3_ perovskite films without and with IMD.


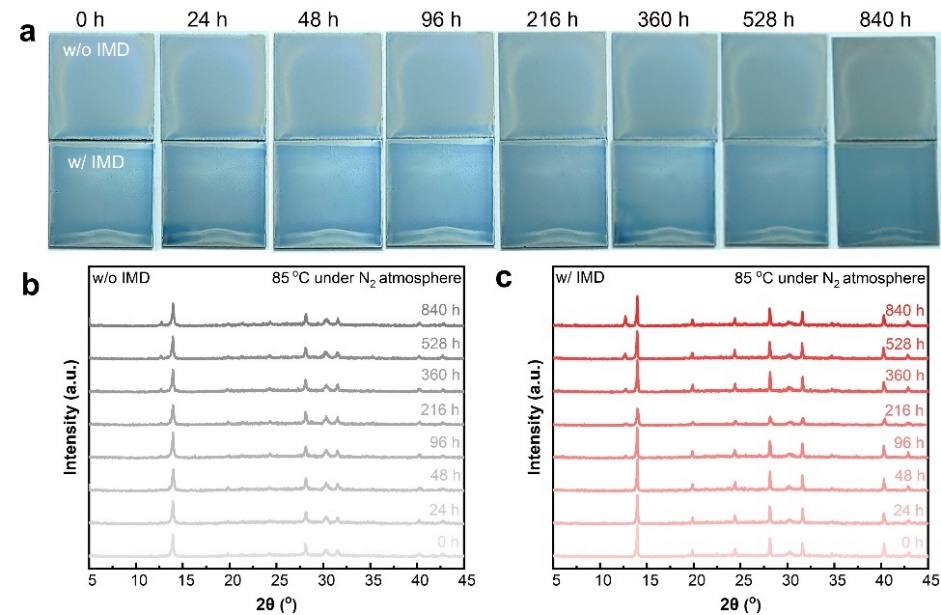


Figure S28. Thermal stability of FAPbI_3_ perovskite films at 85^o^C in the N_2_ without and with IMD.

Table S1. Energies of monomer structure of δ-FAPbI_3_, δ-FAPbI_3_-IMD, α-FAPbI_3_, IMD.

| Structure | Energy (eV) |
| --- | --- |
| δ-FAPbI_3_ | -111.28311 |
| δ-FAPbI_3_-IMD | -182.51617 |
| α-FAPbI_3_ | -110.75774 |
| IMD | -73.051016 |

ΔE1 = E(α-FAPbI_3_) - E(δ-FAPbI_3_) =-110.75774+111.28311= 0.52537 eV

ΔE2 =E (α-FAPbI_3_) + E (IMD) - E(δ-FAPbI_3_-IMD)

=-110.75774-73.051016+182.51617

= -1.292586 eV

Table S2. Summary of devices performances of the reported MA^+^, Cs^+^, Rb^+^ and Br^-^ free α-FAPbI_3_ PSCs.

| Device configuration | Method | Voc (V) | Jsc (mA/cm^2^) | FF (%) | PCE (%) | Area  (cm^2^) | Years |
| --- | --- | --- | --- | --- | --- | --- | --- |
| FTO/C-TiO_2_/FAPbI_3_/P_3_HT/Au | Spin-coating | 0.84 | 18.3 | 50 | 7.5 | 0.15 | 2014^[5]^ |
| FTO/C-TiO_2_/M-TiO_2_/FAPbI_3_/P_3_HT/Au | Spin-coating | 0.73 | 19.24 | 54 | 7.51 | 0.09 | 2014^[6]^ |
| FTO/C-TiO_2_/FAPbI_3_/Spiro-OMeTAD/Au | Spin-coating | 0.94 | 23.3 | 65 | 14.2 | 0.0625 | 2014^[7]^ |
| FTO/C-TiO_2_/M-TiO_2_/FAPbI_3_/Spiro-OMeTAD/Ag | Spin-coating | 1.04 | 18.9 | 68 | 13.7 | 0.09 | 2015^[8]^ |
| FTO/C-TiO_2_/FAPbI_3_/Spiro-OMeTAD/Ag | Spin-coating | 1.00 | 23.65 | 74 | 17.50 | 0.05 | 2015^[9]^ |
| FTO/C-TiO_2_/FAPbI_3_/Spiro-OMeTAD/Ag | Spin-coating | 1.09 | 20.4 | 61.9 | 13.8 | 0.12 | 2016^[10]^ |
| FTO/C-TiO_2_/FAPbI_3_/Spiro-OMeTAD/Ag | Spin-coating | 1.057 | 23.20 | 70.9 | 17.36 |  | 2017^[11]^ |
| FTO/ SnO_2_/FAPbI_3_/Spiro-OMeTAD/Au | Spin-coating | 1.11 | 21.5 | 75.7 | 18 | 0.121 | 2020^[12]^ |
| ITO/NiOx/FAPbI_3_/PCBM/BCP/Ag | blade coating | 1.04 | 23.29 | 76 | 18.41 | 0.09 | 2020^[13]^ |
| FTO/SnO_2_/FAPbI_3_/Spiro-OMeTAD/Au | Spin-coating | 1.04 | 24.8 | 74.6 | 19.3 | 0.11 | 2020^[14]^ |
| FTO/SnO_2_/FAPbI_3_/Spiro-OMeTAD/Au | Spin-coating | 1.165 | 24.4 | 81.3 | 23.1 | 0.16 | 2020^[15]^ |
| FTO/C-TiO_2_/M-TiO_2_/FAPbI_3_/Spiro-OMeTAD/Au | Spin-coating | 1.31 | 23.94 | 77.3 | 20.92 | 0.16 | 2021^[16]^ |
| FTO/SnO_2_/FAPbI_3_/Spiro-OMeTAD/Ag | Spin-coating | 1.10 | 24.79 | 81.5 | 22.22 | 0.125 | 2021^[17]^ |
| ITO/ SnO_2_/FAPbI_3_/Spiro-OMeTAD/Au | Thermal  evaporation | 1.07 | 24.9 | 75.4 | 20.19 | 0.1 | 2022^[18]^ |
|  |  | 1.064 | 24.13 | 73.6 | 18.91 | 1.0 |  |
| ITO/PTAA/FAPbI_3_/PCBM/BCP/Au | Spin-coating | 1.10 | 25.50 | 77 | 21.6 |  | 2022^[19]^ |
| FTO/SnO_2_/FAPbI_3_/Spiro-OMeTAD/MoO_3_/Ag | Spin-coating | 1.06 | 25.90 | 76.45 | 21.05 | 0.05 | 2023^[20]^ |
| ITO/ SnO_2_/FAPbI_3_/Spiro-OMeTAD/MoO_3_/Au | Spin-coating | 1.14 | 25.99 | 78.15 | 23.15 | 0.050 | 2024^[21]^ |
|  |  | 6.61 | 3.76 | 74.18 | 18.43 | 15.40 |  |
|  | Blade-coating | 6.56 | 3.64 | 73.12 | 17.47 | 15.40 |  |
| ITO/DMAcPA/Al_2_O_3_/PEABr/FAPbI_3_  /PEABr/C_60_/SnO_2_/Ag | Blade-coating | 1.12 | 24.85 | 83.29 | 23.14 | 0.093 | Our work |
|  |  | 6.69 | 4.13 | 70.87 | 19.66 | 12.60 |  |

- **REFERENCES**

[1] G. Kresse, D. Joubert, *Phys. Rev. B* **1999**, *59* (3), 1758.

[2] W. Kohn, L. J. Sham, *Phys. Rev.* **1965**, *140* (4A), A1133.

[3] J. P. Perdew, J. A. Chevary, S. H. Vosko, K. A. Jackson, M. R. Pederson, D. J. Singh, C. Fiolhais, *Phys. Rev. B* **1992**, *46* (11), 6671.

[4] K. Momma, F. Izumi, *Journal of Applied Crystallography* **2011**, *44* (6), 1272.

[5] S. Pang, H. Hu, J. Zhang, S. Lv, Y. Yu, F. Wei, T. Qin, H. Xu, Z. Liu, G. Cui, *Chem. Mater.* **2014**, *26* (3), 1485.

[6] S. Lv, S. Pang, Y. Zhou, N. P. Padture, a. L. W. Hao Hu, X. Zhou, H. Zhu, L. Zhang, C. Huanga, G. Cui, *Phys. Chem. Chem. Phys.* **2014**, *16* (36), 19206.

[7] G. E. Eperon, S. D. Stranks, C. Menelaou, M. B. Johnston, L. M. Herz, H. J. Snaith, *Energy Environ. Sci.* **2014**, *7* (3), 982.

[8] Z. Wang, Y. Zhou, S. Pang, Z. Xiao, J. Zhang, W. Chai, H. Xu, Z. Liu, N. P. Padture, G. Cui, *Chem. Mater.* **2015**, *27* (20), 7149.

[9] F. Wang, H. Yu, H. Xu, N. Zhao, *Adv. Funct. Mater.* **2015**, *25* (7), 1120.

[10] Y. Zhou, M. Yang, J. Kwun, O. S. Game, Y. Zhao, S. Pang, N. P. Padture, K. Zhu, *Nanoscale* **2016**, *8* (12), 6265.

[11] G. Li, T. Zhang, F. Xu, Y. Zhao, *Mater. Today Energy* **2017**, *5*, 293.

[12] S. Masi, C. Echeverría-Arrondo, K. M. M. Salim, T. T. Ngo, P. F. Mendez, E. López-Fraguas, D. F. Macias-Pinilla, J. Planelles, J. I. Climente, I. n. Mora-Seró, *ACS Energy Lett.* **2020**, *5* (2), 418.

[13] W. He, J. Hu, C. Chen, Y. Chen, L. Zeng, X. Zhang, B. Cai, Y. Mai, F. Guo, *ACS Appl. Mater. Interfaces* **2020**, *12* (50), 55830.

[14] S. K. Yadavalli, Z. Dai, M. Hu, Q. Dong, W. Li, Y. Zhou, R. Zia, N. P. Padture, *Acta Mater.* **2020**, *193*, 10.

[15] H. Lu, Y. Liu, P. Ahlawat, A. Mishra, W. R. Tress, F. T. Eickemeyer, Y. Yang, F. Fu, Z. Wang, C. E. Avalos, B. I. Carlsen, A. Agarwalla, X. Zhang, X. Li, Y. Zhan, S. M. Zakeeruddin, L. Emsley, U. Rothlisberger, L. Zheng, A. Hagfeldt, M. Grätzel, *Science* **2020**, *370* (6512), eabb8985.

[16] X. Ling, H. Zhu, W. Xu, C. Liu, L. Pan, D. Ren, J. Yuan, B. W. Larson, C. Grӓtzel, A. R. Kirmani, O. Ouellette, A. Krishna, J. Sun, C. Zhang, Y. Li, S. M. Zakeeruddin, J. Gao, Y. Liu, J. R. Durrant, J. M. Luther, W. Ma, M. Grätzel, *Angew. Chem. Int. Ed.* **2021**, *60* (52), 27299.

[17] Y. Zhang, Y. Li, L. Zhang, H. Hu, Z. Tang, B. Xu, N.-G. Park, *Adv. Energy Mater.* **2021**, *11* (47), 2102538.

[18] D. Lin, Y. Gao, T. Zhang, Z. Zhan, N. Pang, Z. Wu, K. Chen, T. Shi, Z. Pan, P. Liu, W. Xie, *Adv. Funct. Mater.* **2022**, *32* (48), 2208392.

[19] T. Du, T. J. Macdonald, R. X. Yang, M. Li, Z. Jiang, L. Mohan, W. Xu, Z. Su, X. Gao, R. Whiteley, C.-T. Lin, G. Min, S. A. Haque, J. R. Durrant, K. A. Persson, M. A. McLachlan, J. Briscoe, *Adv. Mater.* **2022**, *34* (9), e2107850.

[20] D. Wang, M. Chen, X. Zhang, L. Chao, T. Niu, Y. Lv, G. Xing, Y. Xia, M. Li, H. Zhang, Y. Chen, *ACS Appl. Mater. Interfaces* **2023**, *15* (13), 16818.

[21] T. Niu, L. Chao, Y. Xia, K. Wang, X. Ran, X. Huang, C. Chen, J. Wang, D. Li, Z. Su, Z. Hu, X. Gao, J. Zhang, Y. Chen, *Adv Mater* **2024**, e2309171.
